# Investigating sources of inaccuracy in wearable optical heart rate sensors

**DOI:** 10.1038/s41746-020-0226-6

**Published:** 2020-02-10

**Authors:** Brinnae Bent, Benjamin A. Goldstein, Warren A. Kibbe, Jessilyn P. Dunn

**Affiliations:** 1 Department of Biomedical Engineering, Duke University, Durham, NC USA; 2 Department of Bioinformatics and Biostatistics, Duke University, Durham, NC USA

**Keywords:** Biomedical engineering, Imaging and sensing, Technology

## Abstract

As wearable technologies are being increasingly used for clinical research and healthcare, it is critical to understand their accuracy and determine how measurement errors may affect research conclusions and impact healthcare decision-making. Accuracy of wearable technologies has been a hotly debated topic in both the research and popular science literature. Currently, wearable technology companies are responsible for assessing and reporting the accuracy of their products, but little information about the evaluation method is made publicly available. Heart rate measurements from wearables are derived from photoplethysmography (PPG), an optical method for measuring changes in blood volume under the skin. Potential inaccuracies in PPG stem from three major areas, includes (1) diverse skin types, (2) motion artifacts, and (3) signal crossover. To date, no study has systematically explored the accuracy of wearables across the full range of skin tones. Here, we explored heart rate and PPG data from consumer- and research-grade wearables under multiple circumstances to test whether and to what extent these inaccuracies exist. We saw no statistically significant difference in accuracy across skin tones, but we saw significant differences between devices, and between activity types, notably, that absolute error during activity was, on average, 30% higher than during rest. Our conclusions indicate that different wearables are all reasonably accurate at resting and prolonged elevated heart rate, but that differences exist between devices in responding to changes in activity. This has implications for researchers, clinicians, and consumers in drawing study conclusions, combining study results, and making health-related decisions using these devices.

## Introduction

Wearable technology has the potential to transform healthcare and healthcare research by enabling accessible, continuous, and longitudinal health monitoring. With the number of chronically ill patients and health system utilization in the US at an all-time high,^1,2^ the development of low-cost, convenient, and accurate health technologies is increasingly sought after to promote health as well as improve research and healthcare capabilities. It is expected that 121 million Americans will use wearable devices by 2021.^3^ The ubiquity of wearable technology provides an opportunity to revolutionize health care, particularly in communities with traditionally limited healthcare access.

The growing interest in using wearable technologies for clinical and research applications has accelerated the development of research-grade wearables to meet the needs of biomedical researchers for clinical research and digital biomarker development.^4^ Consumer-grade wearables, in contrast to research-grade wearables, are designed, developed, and marketed to consumers for personal use. While research- and consumer-grade wearables often contain the same sensors and are quite similar functionally, their markets and use cases are different, which may influence accuracy (Supplementary Table 1). Digital biomarkers are digitally collected data that are transformed into indicators of health outcomes. Digital biomarkers are expected to enable actionable health insights in real time and outside of the clinic. Both consumer- and research-grade wearables are frequently being used in research, with the most common brands being Fitbit (PubMed: 476 studies, ClinicalTrials.gov: 449 studies) for consumer-grade wearables and Empatica (PubMed: 22 studies, ClinicalTrials.gov: 22 studies) for research-grade wearables (Supplementary Table 2).

It is, therefore, of critical importance to evaluate the accuracy of the wearable technologies that are being used in clinical research, digital biomarker development, and personal health. The lack of clarity surrounding the verification and validation procedures and the unknown reliability of the data generated by these wearable technologies poses significant challenges for their adoption in research and healthcare applications.^4–6^

Recently, the accuracy of wearable optical heart rate (HR) measurements using photoplethysmography (PPG) has been questioned extensively.^7–13^ Wearables manufacturers sometimes report some expected sources of error, but the reporting and evaluation methods are inconsistent^14–22^ (Table 1). Of particular interest, previous research demonstrated that inaccurate PPG HR measurements occur up to 15% more frequently in dark skin as compared to light skin, likely because darker skin contains more melanin which absorbs more green light than lighter skin.^23–31^ Interestingly, some manufacturers of wearable devices recommend using their device only in light skin tones and/or at rest.^17,32^

**Table 1:** Reported accuracy, outliers, evaluation process, and factors that affect performance by each device manufacturer.

Company	Reported accuracy/outliers	Reported evaluation process	Reported factors that affect performance
APPLE	For a small percentage of users, various factors may make it impossible to get any heart rate reading at all^13^	–	Skin perfusion, tattoos, rhythmic movements^14^
FITBIT	–	5000 + hours of activity, exercise, and sleep to iterate through their heart rate technology and that they have over 50 prototype iterations since 2010^15^	–
GARMIN	Skin tone may affect heart rate accuracy but “Garmin designs our watches to work on all skin tones… the sensor may have to work harder [when more melanin is present in the skin] to find the pulse which can require slightly more battery power”^16^	-	Wearing a watch too tightly, participating in activities that cause flexing of the wrist, tattoos^16,17^
XIAOMI	–	–	–
EMPATICA	–	Provides information about algorithms used to calculate HR but not evaluation^18^	–
BIOVOTION	HR is within ±5 bpm under motion. Mean absolute difference (MAD) = 3 bpm and mean absolute relative difference (MARD) = 3% under motion^20^	The proprietary algorithms of the Everion are constantly tested and evaluated in our Algorithmics Lab. Biovotion is dedicated to delivering high quality and accuracy data to empower consumers to take control of their health. At Biovotion everybody is testing the devices under all kinds of conditions and we are working hard to improve the algorithms^19^	Skin perfusion, tattoos, motion^21^

Device manufacturers sometimes report some expected sources of error, but the reporting and evaluation methods are inconsistent,^14–22^ as shown in this table.

Another suspected measurement error in wrist-worn devices is motion artifact, which is typically caused by displacement of the PPG sensor over the skin, changes in skin deformation, blood flow dynamics, and ambient temperature.^33,34^ Motion artifacts may manifest as missing or false beats which result in incorrect HR calculations.^35–37^ Several studies have demonstrated that HR measurements from wearable devices are often less accurate during physical activity or cyclic wrist motions.^8,11,35,38,39^ Several research groups and manufacturers have identified that cyclical motion can affect accuracy of HR in wearable sensors.^9,10,15^ The cyclical motion challenge has been described as a “signal crossover” effect wherein the optical HR sensors on wearables tend to lock on to the periodic signal stemming from the repetitive motion (e.g., walking and jogging) and mistake that motion as the cardiovascular cycle.^40^

To date, no studies have systematically validated wearables under various movement conditions across the complete range of skin tones, and particularly on skin tones at the darkest end of the spectrum. Here, we present a comprehensive analysis of wearables HR measurement accuracy during various activities in a group of 53 individuals equally representing all skin tones. To our knowledge, this is the first reported characterization of wearable sensors across the complete range of skin tones. Validation of wearable devices during activity and across all skin tones is critical to enabling their equitable use in clinical and research applications.

## Results

### Study summary

**Fig. 1:**
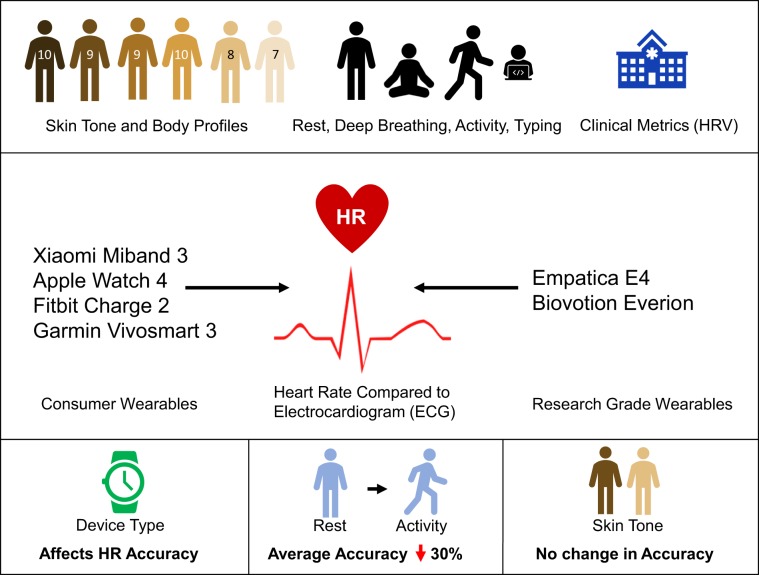
Graphical abstract of research. Graphical abstract of research study presented. We present a full characterization of HR accuracy across skin tones, clinical metrics of HRV accuracy across skin tones, and HR during activity, rest, deep breathing, and typing for six wearable devices representing both consumer wearables and research-grade wearables. HR metrics are compared to the clinical-grade electrocardiogram (ECG) as the standard for heart rate measurement.

A group of 53 individuals successfully completed the entire study protocol (32 females, 21 males; ages 18–54; equal distribution across the Fitzpatrick (FP) skin tone scale). This protocol was designed to assess error and reliability in a total of six wearable devices (four consumer-grade and two research-grade models) over the course of approximately 1 h (Fig. 1). Each round of the study protocol, included (1) seated rest to measure baseline (4 min), (2) paced deep breathing^41^ (1 min), (3) physical activity (walking to increase HR up to 50% of the recommended maximum;^42^ 5 min), (4) seated rest (washout from physical activity) (~2 min), and (5) a typing task (1 min). This protocol was performed three times per study participant in order to test all devices. In each round, the participant wore multiple devices according to the following: Round 1: Empatica E4 + Apple Watch 4; Round 2: Fitbit Charge 2; Round 3: Garmin Vivosmart 3, Xiaomi Miband, and Biovotion Everion. The electrocardiogram (ECG) patch (Bittium Faros 180) was worn during all three rounds. The ECG was used as the reference standard for this study.

Potential relationships between error in HR measurements and (1) skin tone, (2) activity condition, (3) wearable device, and (4) wearable device category were examined using mixed effects statistical models. We developed comprehensive, individual, and interaction mixed effects models for the independent variables using mean HR measurement error as the dependent variable (Table 2). We found that wearable device, wearable device category, and activity condition all significantly correlated with HR measurement error, but changes in skin tone did not impact measurement error or wearable device accuracy.

**Table 2:** Results of mixed effects comprehensive and marginal models.

Mixed effects model	Mean error *p* value (***<0.001)
Comprehensive model	<2.20e−16***
Marginal model: skin tone	0.634
Marginal model: activity condition	<2.20e−16***
Marginal model: device	<2.20e−16***
Marginal model: type of device	3.44e−05***
Interaction model: skin tone and device	2.80e−05***

*p* Values show results of likelihood ratio tests between models and null models and interaction models.

### Wearables accuracy across skin tones

Anecdotal evidence and incidental study findings supported the hypothesis that PPG measurements may be less accurate on darker skin tones than on lighter skin tones.^8–13^ To systematically explore this hypothesis, we examined the mean directional error (MDE) and the mean absolute error (MAE) of HR measurements within each FP skin tone group at rest and during physical activity.

**Fig. 2:**
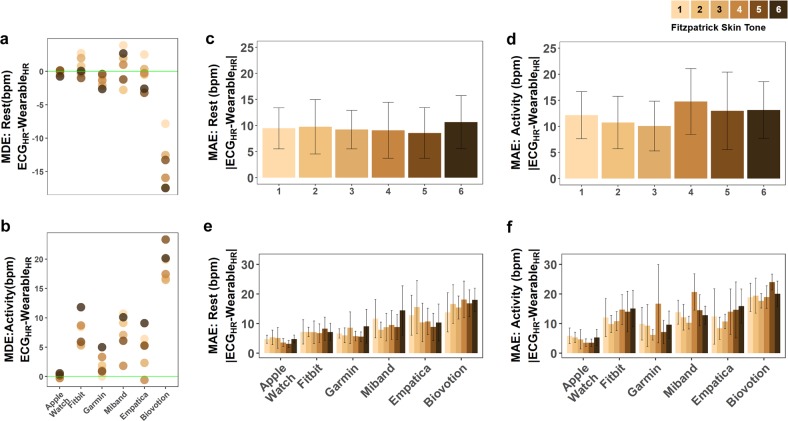
Error in heart rate across skin tones and devices at rest and during activity. Mean error in heart rate (bpm) across skin tones and devices at **a** rest and **b** during physical activity. The green horizontal line represents no error (no difference from the true measurement of HR from ECG). Mean absolute error in heart rate (bpm) across skin tones and devices at **c** rest and **d** during physical activity. Error is calculated as the difference between the ECG and wearable reported heart rate at every simultaneous measurement. Fitzpatrick skin tones 1–6 are represented with an approximately equal number of participants in each skin tone. Error bars represent the 95% confidence interval. Mean absolute error across devices and across skin tones at rest (**e**) and during activity (**f**). Error bars represent the 95% confidence interval.

Among skin tone groups at rest, FP5 had the largest MDE across all devices and FP1 had the lowest MDE (−4.25 bpm and −0.53 bpm, respectively) (Supplementary Figs 1a, 2a, Supplementary Table 7a). In absolute error terms, the darkest skin tone (FP6) had the highest MAE and the second darkest skin tone (FP5) had the lowest MAE at rest (10.6 bpm and 8.6 bpm, respectively) (Fig. 2c, e, Supplementary Table 6a). The average MDE and MAE across all skin tone groups at rest were −2.99 bpm and 9.5 bpm, respectively. Among skin tone groups during activity, FP5 had the highest MDE and FP3 had the lowest MDE (9.21 bpm and 7.21 bpm, respectively; Fig. 2b, Supplementary Table 7b). FP4 had the highest MAE and FP3 had the lowest MAE (14.8 bpm and 10.1 bpm, respectively; Fig. 2d, f, Supplementary Table 6b). Skin tone appears to not be the driver of MAE or MDE.

In the comprehensive and marginal mixed effects models, we found no significant correlation between skin tone and HR measurement error (Table 2). While we found no overall effect of skin tone, we tested whether the effect of skin tone differed based on individual devices. We did find a significant interaction between skin tone and device (Table 2). Upon further examination, this was shown to be based on the Biovotion device, which showed a decrease in resting HR and increase active HR (Fig. 2). During activity, the highest MDE occurs in FP5 and/or FP6 in all devices except for the Xiaomi Miband 3 (Fig. 2b).

We also explored whether there were differences in data loss for the different skin tone groups. Some measurement circumstances may prevent data acquisition altogether, such as when a device is not making contact with the skin. In other cases, wearables appear to remove data that fails internal quality control, for example, when there is a large motion artifact (indicated by high accelerometry sensor values), the device internal quality system may remove the data points potentially affected by the artifact. Both of these scenarios can cause missingness in the data that is reported by the wearable. Because the research-grade devices used in this study use downsampling or interpolation to provide data at exactly 1 Hz, they were inappropriate to include in the data missingness analysis. Missingness was calculated as the percent of values that are missing based on the expected sampling rate (here, the average sampling rate over the course of the study). The missingness analysis showed no significant difference between skin tones (Supplementary Table 8).

In addition to HR, we examined HR variability (HRV), a clinically relevant diagnostic metric that can be derived from PPG signals and is a widely used metric of autonomic nervous system function.^43^ The standard HRV metrics we examined, included the time-domain metrics, included mean HRV, minimum HRV, maximum HRV, RMSSD, SDNN, and pNN50 (Supplementary Fig. 4). No differences were seen in accuracy of the HRV metrics between the different skin tone groups (two-sided, unpaired *t* test between skin tones, Bonferroni-corrected *p* = 0.0033).

### Wearables accuracy at rest and during physical activity

**Fig. 3:**
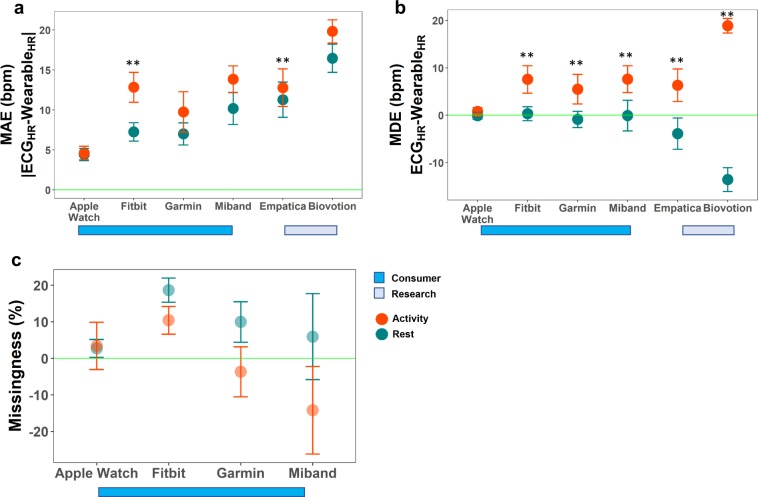
Error in heart rate across all devices and analysis of missing values across consumer devices. **a** Mean absolute error in heart rate (bpm) across devices during rest (teal) and activity (orange). This shows the true difference in HR from the ECG but does not show the sign of the difference. The green horizontal line represents no error (no difference from the true measurement of HR from ECG). Error bars show the 95% confidence interval. ** indicates significant difference in error between baseline and activity with a Bonferroni multiple hypothesis corrected *p* value of 0.0042. **b** Mean relative error in heart rate (bpm) across devices during rest (teal) and during activity (orange) shows the relative differences from the ECG. The green horizontal line represents no error (no difference from the true measurement of HR from ECG). Error bars show the 95% confidence interval. ** indicates significant difference in error between baseline and activity with a Bonferroni multiple hypothesis corrected *p* value of 0.0042. **c** Analysis of missing values across skin tones for rest and activity for consumer wearables. Research-grade wearables (Empatica, Biovotion) down-sample and/or interpolate to have exactly 1 Hz sampling rate and thus we could not calculate missingness values for those devices. Missingness is calculated from the expected sampling rate (reported sampling rate for Apple Watch and Garmin and study average sampling rate for Garmin and Miband, which do not report sampling rate). Missingness that is positive indicates percentage of values with missingness. Missingness that is negative indicates a greater than expected sampling rate (more values than expected).

Consumer-grade wearables were found to be more accurate than research-grade wearables at rest. During rest, the MAE (±standard deviation (SD)) of consumer wearables was an average of 7.2 ± 5.4 bpm and the MAE of research-grade wearables was 13.9 ± 7.8 bpm (*p* < 0.0125). During physical activity, the MAE ± SD of consumer wearables was 10.2 ± 7.5 bpm and the MAE of research-grade wearables was 15.9 ± 8.1 bpm (*p* < 0.0125) (Fig. 3a; Supplementary Table 3). While these devices, separated by either research- or consumer-grade categories, showed significant differences in accuracy based on the mixed effects models, it is important to note that the major drivers behind this significance were the Apple Watch (consumer-grade device) and Biovotion Everion (research-grade device).

Among the consumer wearables tested at rest, the Xiaomi Miband 3 had the highest MAE and the Apple Watch 4 had the lowest MAE (10.2 bpm vs. 4.4 bpm, respectively) (Fig. 3a). Among the research wearables tested at rest, the Biovotion Everion had the highest MAE and the Empatica E4 had the lowest MAE (16.5 bpm and 11.3 bpm, respectively) (Fig. 3a). Consistency in accuracy for devices was evaluated by comparing the standard deviation of the MAE among consumer-grade and research-grade devices at rest. At rest, the standard deviation of the MAE was highest for the Fitbit Charge 2 and lowest for Apple Watch 4 (7.3 and 2.7 bpm, respectively). In research wearables at rest, the standard deviation of the MAE was highest for the Empatica E4 and lowest for Biovotion Everion (8.0 and 6.4 bpm, respectively).

This demonstrates that both the accuracy and the consistency of HR measurements varies by device model (Fig. 3a, Supplementary Table 3), and that the measurements from the research-grade wearables were less accurate than measurements from the consumer-grade wearables.

During physical activity among consumer-grade devices, the Xiaomi Miband 3 had the highest MAE and the Apple Watch 4 had the lowest MAE (13.8 bpm and 4.6 bpm, respectively). The Garmin had the highest standard deviation of MAE and the Apple Watch 4 had the lowest standard deviation of MAE among consumer-grade devices (9.2 and 3.0, respectively). Among research-grade devices during physical activity, the Biovotion had the highest MAE and the Empatica E4 had the lowest MAE (19.8 bpm and 12.8 bpm, respectively) and the Empatica E4 had the highest standard deviation of MAE and the Biovotion Everion had the lowest standard deviation of MAE (8.5 and 5.3, respectively). This further demonstrates that the consistency and accuracy also vary by device type during physical activity (Fig. 3a, Supplementary Table 3).

Interestingly, at rest, the MDE of the research-grade devices was negative, indicating that the reported HR from the research-grade wearables was lower than the true HR (Fig. 3b, Supplementary Table 4). The positive values of MDE during physical activity for both consumer- and research-grade devices demonstrate that HR values reported by wearables are higher than the true HR values during physical activity (Fig. 3b). Overall, the MDE increased significantly in the positive direction between rest and physical activity for 5 out of 6 devices (Fig. 3b, Supplementary Table 4).

We also examined missingness for consumer-grade devices during physical activity as compared with rest. Again, this analysis was not relevant for research-grade devices based on their downsampling/interpolation methods. At rest, missingness was highest for the Fitbit Charge 2 and lowest for the Apple Watch 4 (18.7 and 2.7%, respectively). During physical activity, missingness was highest for the Fitbit Charge 2 and lowest for the Xiaomi Miband 3 (10.4 and −14.2%, respectively). Here, a negative value for percent missingness occurs because the device samples at a higher sampling rate than expected during certain time periods. We found that both missingness and overall accuracy were mostly unchanged between rest and activity for the Apple Watch. The variability of missingness was highest for the Xiaomi Miband 3 and lowest for the Apple Watch 4 during rest (SD of 42.7% and 9.0%, respectively), and was highest for the Xiaomi Miband 3 and lowest for the Fitbit Charge 2 during physical activity (SD of 43.5% and 13.7%, respectively). As shown in Fig. 3c, all devices except for the Apple Watch 4 had lower missingness during physical activity, indicating that they likely sample at higher sampling rates during activity than at rest (Fig. 3c, Supplementary Table 5).

### Wearables accuracy during rhythmic activity

Periodic, rhythmic movement has been cited as a source of error in optical HR measurements in previous studies.^8,44,45^ This has been described as a “signal crossover” effect wherein the optical HR sensors on wearables tend to lock on to the periodic signal stemming from the repetitive motion (e.g., walking and jogging) and mistake that signal as the cardiovascular cycle.^40^ We show that the rhythmic movement of walking has significantly higher errors in all devices except the Apple Watch 4 (Fig. 3b). We also explored repetitive wrist motion involved in typing (Supplementary Fig. 3) and found that MAE was higher during typing compared with rest in all devices, and often nearly as high as during walking, except for the Apple Watch and the Empatica E4 (Supplementary Fig. 3a). The MDE was higher during typing as compared with rest in the Miband, Empatica, and Biovotion. Interestingly, while both typing and walking had poor performance overall, walking tended to cause reported HR to be higher than true HR, whereas typing caused the reported HR to be lower than the true HR (Supplementary Fig. 3b). Surprisingly, the MAE and MDE were lower during deep breathing than at rest in all devices except for the Apple Watch, in which the deep breathing condition was the condition with the worst performance (Supplementary Fig. 3). During deep breathing, reported HR was generally lower than true HR (Supplementary Fig. 3).

### Signal alignment

Lags between the ECG- and PPG-derived HR signals ranging between 0 and 43 s were discovered during our preliminary exploratory data analysis. These lags were inconsistent; in some cases, the lag was fixed and in other cases the lag was dynamic (Supplementary Fig. 5). The source of these lags could not be pinned down with certainty and may possibly be attributed to (1) misaligned time stamps (highly unlikely due to our time synchronization protocol described in the methods as well as the sometimes dynamic time lags observed), (2) data processing artifacts (uneven or delayed sampling, compute, and/or data reporting), (3) missed heart beats due to low frequency measurements by the wearable, or (4) a delay between the actual heart beat and the change in blood volume at wrist.

In order to remove lag as a factor that could contribute to error calculated in the previous sections, we performed signal alignment using two different approaches (cross-correlation and smoothing with a rolling window) and recalculated MAE and MDE on the newly aligned signals (Supplementary Fig. 6). Using the updated MAE and MDE at each window size from the smoothing, we reanalyzed the relationships in the previous sections and found no differences in conclusions from the previous sections. Our model did show that window length is related to HR measurement error (Supplementary Table 9). We performed a sensitivity analysis to determine how smoothing could affect improvements in accuracy, and we found that in most cases, smoothing reduced HR measurement error as demonstrated by the fact that the median optimal window size >0 (Supplementary Fig. 7). MAE and MDE were in general improved the most by smaller window sizes (less smoothing) during activity and wider window sizes (more smoothing) at rest, likely because changes in activity intensity would not be captured by wider smoothing windows. (Supplementary Fig. 7b). This did not hold true for the Apple Watch 4 and Empatica E4 for MDE or the Biovotion Everion for MAE.

### Potential relationship between wearable device cost, market size, release year, and error

Wearables vary widely in terms of release year, data accessibility, and cost (Supplementary Table 1). We used devices across a wide range of costs, market sizes, and release times at the time of this study (Apple Watch 4, Fitbit Charge 2, Garmin Vivosmart 3, and Xiaomi Miband 3; cost range = $432 USD, 2018 market size range = 35.7 million; Supplementary Table 1). In general, we found that devices with higher cost, a more recent release date, and a larger market had higher accuracy. Because of the limited scope of the devices used, we cannot tease apart the effects of each of these three factors. While device release year is noted here, all devices used in this study had software updates as of the beginning of the study. Thus, while hardware differences may exist, software is updated frequently on these devices to help prevent obsolescence in the older technologies.

## Discussion

The rise in accessibility of consumer wearable devices that generate health information provides an unprecedented opportunity to revolutionize health care by researchers, clinicians, and consumers. In this study, we aimed to determine whether there were differences in wearable device accuracy across (1) skin tones, (2) activity conditions, and (3) devices, in order to analyze potential measurement errors. This is the first time that skin tone has been comprehensively explored as a potential factor affecting HR accuracy. Anecdotal evidence and incidental study findings previously indicated that wearable HR measurements may be less accurate in darker skin tones due to higher absorption of light at the typical wearable green light wavelength. Overall, we did not find statistically significant differences in HR or HRV accuracy across skin tones.

Our analyses reinforces the growing body of research demonstrating that wearable devices have higher error during activity than at rest.^8,11,35,38,39^ We further demonstrate that the directionality of the HR error is dependent on the activity type. Reliable HR data across all activity types and levels is key to enabling digital biomarker development and to supporting clinical research studies that involve physiologic monitoring during physical activity or exercise interventions. It is also critically important to many health consumers who use devices to ensure that they do not exceed their maximum HR during exercise, which is a circumstance that can spur adverse cardiac events.^46^

Here, we demonstrate an overall over-reporting of HR during low-intensity physical activity, which could be a safety mechanism engineered into the consumer devices to account for error so that consumers do not exceed their maximum HR during exercise. This is especially relevant for clinicians, who should be aware of these biases in HR measurements during exercise when making clinical assessments based on HR data from wearable devices. This could affect exercise interventions that are based off HR feedback; accordingly, clinicians may examine alternative ways of measuring exercise intensity if measurement specificity is critical.

While the research-grade wearables are the only wearables that provide users with raw data that can be used to visualize PPG waveforms and calculate HRV, the HR measurements tended to be less accurate than consumer-grade wearables. This is especially important for researchers and clinicians to be aware of when choosing devices for clinical research and clinical decision support. It is our hope that this analysis framework can act as a guide for researchers, clinicians, and health consumers to evaluate such tradeoffs when exploring potential wearable devices for use in a clinical study, digital biomarker development, clinical practice, or in personal health monitoring.

Wearable technologies are expected to transform healthcare through inexpensive and convenient health monitoring outside of the clinic.^4^ This provides an opportunity to bring equitable healthcare access to traditionally underserved communities which can address socioeconomic and racial disparities that exist in the US healthcare system.^47^ Here, we explored one important aspect regarding the accuracy of wearables across the full range of skin tones. We found no statistically significant differences in wearable HR measurement accuracy across skin tones, however, we did find other sources of measurement inaccuracies, including activity type and type of device. Researchers, clinicians, and health consumers must recognize that the information derived from different wearables should not be weighted equally for drawing study conclusions, combining study results, and making health-related decisions. Algorithms that are used to calculate digital biomarkers should consider error and measurement quality under the various circumstances that we have shown in this study. Digital biomarker interpretation must take this data quality into account when making healthcare decisions.

## Methods

### Study population

Totally, 56 participants (34 females, 22 males, 18–54 years of age, mean = 25.6, racial breakdown: 8 African American, 21 Asian, 8 Hispanic, and 19 Caucasian-White) were recruited for this study. Data from three participants was excluded from the study due to incomplete ECG records. The subjects all consented to the study and were compensated for their participation. The study was approved by the Institutional Review Board at Duke University and informed consent was obtained from all participants. We enrolled an approximately equal distribution of skin tones (F1:7, F2:8, F3:10, F4: 9. F5: 9, F6:10) on the FP skin tone scale, the standard skin tone scale with six categories of pigmentation^48,49^ (one to six, one being the lightest and six being the darkest). Participants were excluded if they had skin conditions or sensitivities that would be exacerbated by wearing a wearable device/sensor and/or electrode pads or if they were taking medications/substances that affect HR (including, but not limited to Adderall, performance enhancing drugs, human growth hormones, and illegal substances). The demographics from this study are shown in Supplementary Table 10.

### Sample size

Based on our power analysis, we required ≥48 participants to achieve 80% power to reject the null hypothesis that there is no difference in PPG accuracy between skin tone groups (∝ = 0.5). Effect size for the power analysis was based on a pilot study examining differences in light absorption across skin tones^25^ and was determined to be 0.3. Difference in green light mean absorption for different skin tones during activity was used to calculate effect size since optical HR measurements primarily measure green light absorption.^24,25^ Based on the ANOVA power calculation, we required eight participants per skin tone category (6 skin tone categories on FP scale). We also performed a multiple regression power calculation (f2 = 0.15, power = 0.8, ∝ = 0.5) and determined the number of participants required was a total of 46 for the mixed effects model.

### Devices and data collection protocol

We tested four consumer wearable devices used frequently in research studies, as shown in Fig. 1, including the Apple Watch 4 (Apple Inc., Cupertino, CA), Fitbit Charge 2 (Fitbit, Inc., San Francisco, CA), Garmin Vivosmart 3 (Garmin Ltd., Olathe, Kansas), Xiaomi Miband 3 (Xiaomi Corp., Beijing, China) as well as two research-grade wearable devices Empatica E4 (Empatica Inc., Milano, Italy), and Biovotion Everion (Biovotion AG, Zurich, Switzerland). Device release date and software version are summarized in Supplementary Table 2. All devices were sampled using the highest sampling rate possible (this was done by placing devices in “activity mode” for the duration of the study, when applicable). All devices use PPG to generate HR readings. The wearable device HR readings were compared against HR values calculated from simultaneously measured electrocardiogram (ECG) (Fig. 1). ECG is a clinical-grade gold standard for HR measurement which measures the electrical activity of the heart (Bittium Faros 180, Bittium Inc., Oulu, Finland).

Measured factors included skin tone, gender, body fat percentage, weight, height, waist circumference, and sun exposure habits, chosen based on scientific literature and anecdotal evidence of their potential effects on wrist-based optical HR sensing accuracy. Subjective analysis of skin tone using the FP skin tone scale (1–6) and the von Luschan skin tone scale (1–36) were taken in addition to an objective measurement of skin tone on the wrist using a spectrophotometer (Linksquare, Stratio Inc., San Jose, CA).

We followed the Consumer Technology Association wearable device validation guidelines to measure PPG at rest (seated in upright position) and with physical activity that increases HR to 50% of the maximum HR (treadmill at 2.5–4 mph).^50^

Data were collected and downloaded directly from the Bittium Faros 180 and the Empatica E4. Wearable devices Garmin, Fitbit, and Biovotion were connected to native apps for data access. Apple Watch and Xiaomi Miband required non-native apps to allow for access to HR data.

The study followed three phases of: baseline, deep breathing, activity, washout, and typing task. During baseline, participants were asked to remain seated in a comfortable position for 4 min. This was followed immediately by a deep breathing exercise, where participants breathed in sync with a 1-min deep breathing video.^51^ Participants then participated in a walking activity for 5 min. Participant HR was monitored during this time to ensure that the participant reached 50% of their maximum HR and did not exceed their maximum HR (220-age). A washout period of approximately 2 min occurred before participants began the typing task to ensure HR had returned to baseline. Participants typed on a mechanical computer keyboard (Dell Model: SK-8115) for 1 min before switching devices to begin the next phase.

In the first phase, the Empatica E4 was placed on the right wrist and the Apple Watch 4 on the left wrist. In the second phase, the Fitbit Charge 2 was placed on the left wrist. During the third phase, the Garmin Vivosmart 3 was placed on the right wrist and the Xiaomi Miband 3 was placed on the left wrist. Participants wore the Biovotion on the upper right arm for all three phases but data from only the last phase (Phase 3) was used in this study.

### Time syncing and signal alignment

All wearable devices were connected to Wi-Fi-only enabled mobile device (smart phone or laptop). In order to prevent desynchronization via internal clock time drift, at the start of each study, each wearable device was connected to a mobile device to synchronize the clock time following ISO 8601.^52–55^ Prior to the start of each study, each mobile device was connected to the network to synchronize their internal clock time via the Network Time Protocol (NTPv4).^54^ Once connected to the Wi-Fi and synchronized, the NTP client updates the mobile device clock approximately every 10 min.^56^

The Apple Watch, Fitbit, Garmin, and Biovotion were connected to the iPhone SE (iOS), the Xiaomi Miband was connected to the Android Samsung Galaxy 4 mobile device, and the Bittium Faros and Empatica E4 were connected to ThinkPad Laptop running Windows 10. iPhones running iOS5 and above automatically syncs to the NTP, and the settings in both Android and Windows 10 were set to ensure automatic NTP syncing upon Wi-Fi connection. Because the wearable devices used in this study were not precision instruments, processing lag times between devices that occur during the NTP sync may affect the device clock time by milliseconds.^57^

### Mixed effects modeling

To assess the impact of various factors and account for repeated measurements on participants we used a mixed-model approach. We first fit a null model shown

1 $$Y_{ij} = \alpha _i + s + c + d + \varepsilon _{ij},$$

where the observations (*Y*) is the Difference between ECG HR and Wearable HR for each participant (*i*) at each timepoint (*j*). *ε*_*ij*_ accounts for the random noise. The random effect parameter *α*_*i*_ accounts for participant-specific differences.

Next, we fit univariable models accounting for skin tone (*s*), condition (*c*) (rest, walking, deep breathing, and typing), and device (*d*), respectively

2 $$Y_{ij} = \alpha _i + s + {\it{\epsilon }},$$

3 $$Y_{ij} = \alpha _i + c + {\it{\epsilon }},$$

4 $$Y_{ij} = \alpha _i + d + {\it{\epsilon }}.$$

We also examined an interaction model to examine whether there is an interaction between skin tone and device factors as shown in Eq. (5)

5 $$Y_{ij} = \alpha _i + s \ast d + {\it{\epsilon }}.$$

We assessed the added value of the factor via a likelihood ratio test, comparing the larger model to the null model. A significant *p* value indicates that the larger model provides a better fit. This is akin to repeated measures ANOVA. We used a *p* value < 0.0125, based on a Bonferroni correction to indicate significance (taking three factors—skin tone, device, and activity condition into account).

### Differences algorithm

Raw ECG was processed using the clinical standard, Kubios HRV Premium (version 3.3) to extract RR intervals and HR. Differences between the ECG and each wearable sensor were calculated at each matched timestamp for each wearable sensor for each participant. Both relative and absolute differences were calculated as shown in Eqs. (6) and (7).

6 $${\rm Directional}\,{\rm difference\!:}\,{\rm HR}_{\rm ECG} - {\rm HR}_{\rm Wearable}.$$

7 $${\rm Absolute}\,{\rm difference\!:}\,{\rm |HR}_{\rm ECG} - {\rm HR}_{\rm Wearable}{|}.$$

### Calculations of error

We have defined error as the difference between HR from the ECG and the wearable sensor. Thus, higher error indicates a larger difference between the wearable sensor and the “true” value from the ECG. Error was compared across skin tones using an unpaired, two-sided *t* test with Welch approximation and Bonferroni multiple hypothesis correction of 0.00028 (considering 6 choose 2 skin tone comparisons—15 skin tone comparisons × 6 devices × 2 conditions—rest and activity).

8 $${\rm Mean}\,{\rm directional}\,{\rm error}_{\rm participant}:\,\frac{{\sum} {\rm HR}_{\rm ECG} - {\rm HR}_{\rm Wearable}}{{\rm Number}_{{\rm matched}\,{\rm timestamps}}}.$$

9 $${\rm Mean}\,{\rm absolute}\,{\rm error}_{\rm participant}:\frac{{\sum} {\left| {\rm HR}_{\rm ECG} - {\rm HR}_{\rm Wearable} \right|}}{{\rm Number}_{{\rm matched}\,{\rm timestamps}}}.$$

### Calculations of Missingness

Missingness is calculated from the expected sampling rate (study average sampling rate). The calculation used to determine Missingness (%) is shown in Eq. (10). Statistical differences between missingness for activity and baseline were calculated using paired, two-sided *t* tests with a Bonferroni multiple hypothesis correction (taking into account four devices, *p* value = 0.0125). Statistical differences between missingness for skin tones were calculated using unpaired, two-sided *t* tests between a skin tone and all other skin tones for each device with a Bonferroni multiple hypothesis corrected *p* value of 0.001 (taking into account four devices, six skin tones, and two conditions).

10 $${\rm{Missingness}\,\left( {\%} \right):100 - \left( {\frac{{\rm{Actual}\# {\rm{Samples}}}}{{Expected\# Samples}}} \right) \ast 100}.$$

### Calculations and analysis of HRV

Because HRV requires access to raw, sample-level data that is not currently provided by most wearables, out of the six devices tested, we were limited to using only the Empatica E4 for the HRV accuracy analysis. HRV time-domain metrics from the Empatica device have been validated against ECG in previous studies.^58–60^ Frequency domain metrics of HRV have not been sufficiently validated on wearable optical HR sensors, thus are excluded from this analysis.

HRV was only calculated during baseline due to motion artifacts affecting the signal. Raw ECG was processed using the clinical standard, Kubios HRV Premium (version 3.3) to extract RR intervals. PPG data from the Empatica E4 device is supplied as both raw PPG (green LED light only) and an inter beat interval (IBI) sequence. The IBI sequence provided by Empatica is obtained from their wristband-integrated processing algorithm that removes incorrect peaks due to noise in the raw PPG signal, which they compute from the red and green LEDs on the device. Red LED PPG signal is not saved or provided and is only used in the calculation of the provided IBI sequence.

We matched raw PPG and IBI sequences and removed data that the Empatica wristband- integrated processing algorithm removed onboard. Our updated PPG signal could then be used to extract IBI sequences for HRV calculations. A Kolmogorev–Zurbenko low pass linear filter (Kolmogorev) and outlier removal was used to mitigate any additional motion artifact not removed by the Empatica processing algorithm. Following the process described by Empatica for determining their IBI sequence, local minima were detected using a rolling minimum detector and the IBI values were calculated as the difference between these local minima values. Outlier capping at 1.5*IQR was performed for each downsampled signal.

All calculations for time-domain HRV were performed with user-defined functions in Python (3.5.2) that were validated using Kubios HRV Premium (version 3.3). Error was calculated between the ECG HRV and the PPG HRV for each participant. Paired, two-sided *t* tests were performed with a significance threshold of Bonferroni-corrected *p* value of 0.0033 (considering 6 choose 2 skin tone comparisons—15 skin tone comparisons for each HRV metric).

### Lag time analysis using a rolling window approach

In order to examine the effect of lag time on our model, we iterated through rolling windows of 5, 10, 20, 30, 40, 50, 60, 90, 120, 150, 180, 210, 240 s for each participant, each device, and each condition (rest or activity). We found the optimal window length of MAE and MDE by determining the window length that minimized the MAE and MDE, respectively. We then repeated the mixed effects model, adding window length as an effect.

### Activity level disparity statistical testing

Mean relative error from ECG for both baseline and activity were recorded for each participant. Mean relative errors across participants were used for a paired, two-sided *t* test with Welch approximation and Bonferroni multiple comparison correction with an initial significance threshold of *p* < 0.05 and a Bonferroni-corrected *p* < 0.0042 (considering six devices and two conditions—rest and activity).

### Reporting summary

Further information on research design is available in the Nature Research Reporting Summary linked to this article.

## Supplementary information

Supplemental Material

NR Reporting Summary

## Data Availability

The data sets generated during and/or analyzed during the current study will be submitted one year from the publication date to the PhysioNet public repository under the title BigIdeasLab_STEP.

## Code availability

Code for the algorithm development, evaluation, and statistical analysis is open source with no restrictions and is available from https://github.com/Big-Ideas-Lab/STEP.
