# Supplementary material for: Investigating sources of inaccuracy in wearable optical heart rate sensors
Source: NPJ Digit Med. 2020 Feb 10;3:18. doi: 10.1038/s41746-020-0226-6 (PMC7010823; doi:10.1038/s41746-020-0226-6)
Supplement: Supplementary file 1 — Supplemental Material [file 41746_2020_226_MOESM1_ESM.pdf]

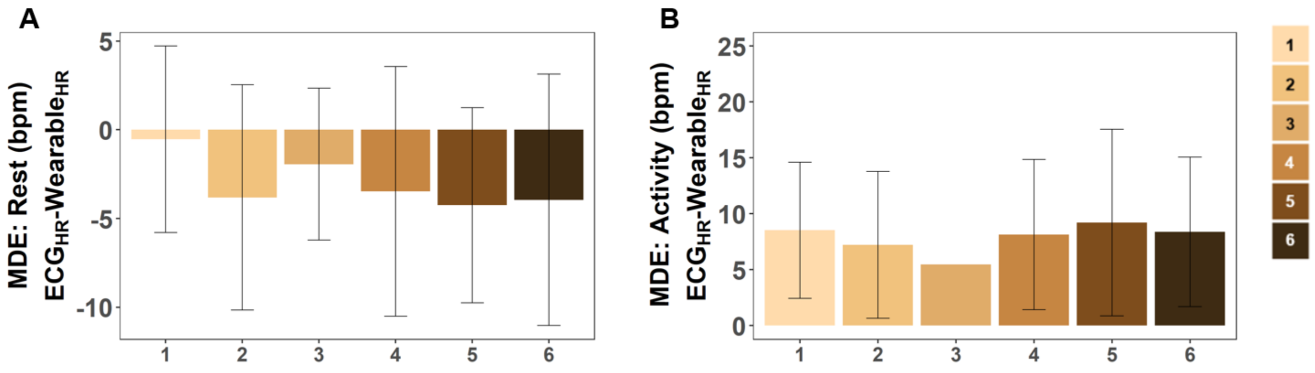

Supplementary Figure 1. *Mean Error in heart rate (bpm) across skin tones and devices at (A) rest and (B) during walking activity. Error is calculated as the difference between the ECG and wearable reported heart rate at every simultaneous measurement. Fitzpatrick skin tones 1-6 are represented with an approximately equal number of participants in each skin tone. Error bars represent the 95% confidence interval.*

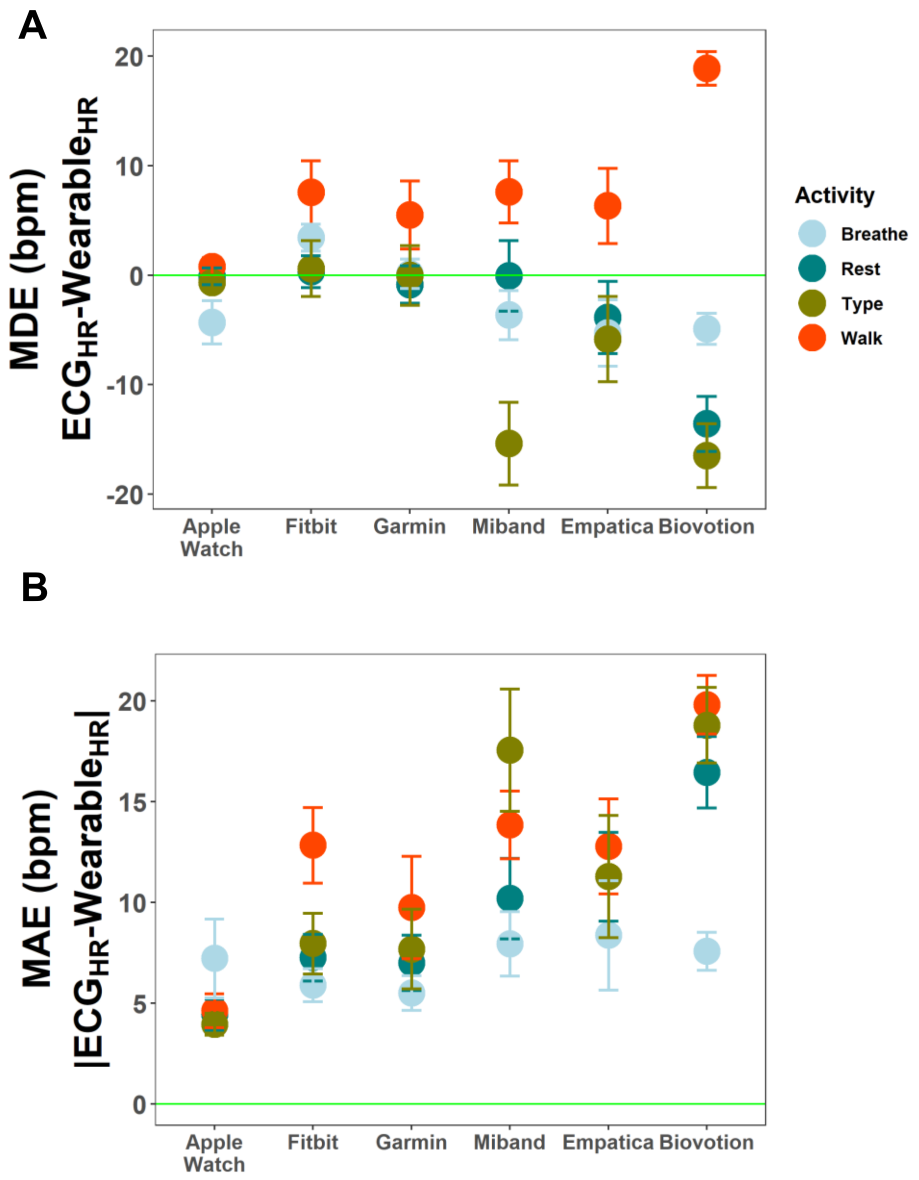

Supplementary Figure 2. (A) *Mean Directional Error in heart rate (bpm) across devices during rest (teal), breathing (light blue), typing (green), and activity (orange) shows the relative differences from the ECG. The green horizontal line represents no error (no difference from the true measurement of HR from ECG). Error bars show the 95% confidence interval.* ***(B)*** *Mean Absolute Error in Heart Rate (bpm) across devices during rest (teal), breathing (light blue), typing (green), and activity (orange). This shows the true difference in HR from the ECG but does not show the sign of the difference. The green horizontal line represents no error (no*

difference from the true measurement of HR from ECG). Error bars show the 95% confidence interval.

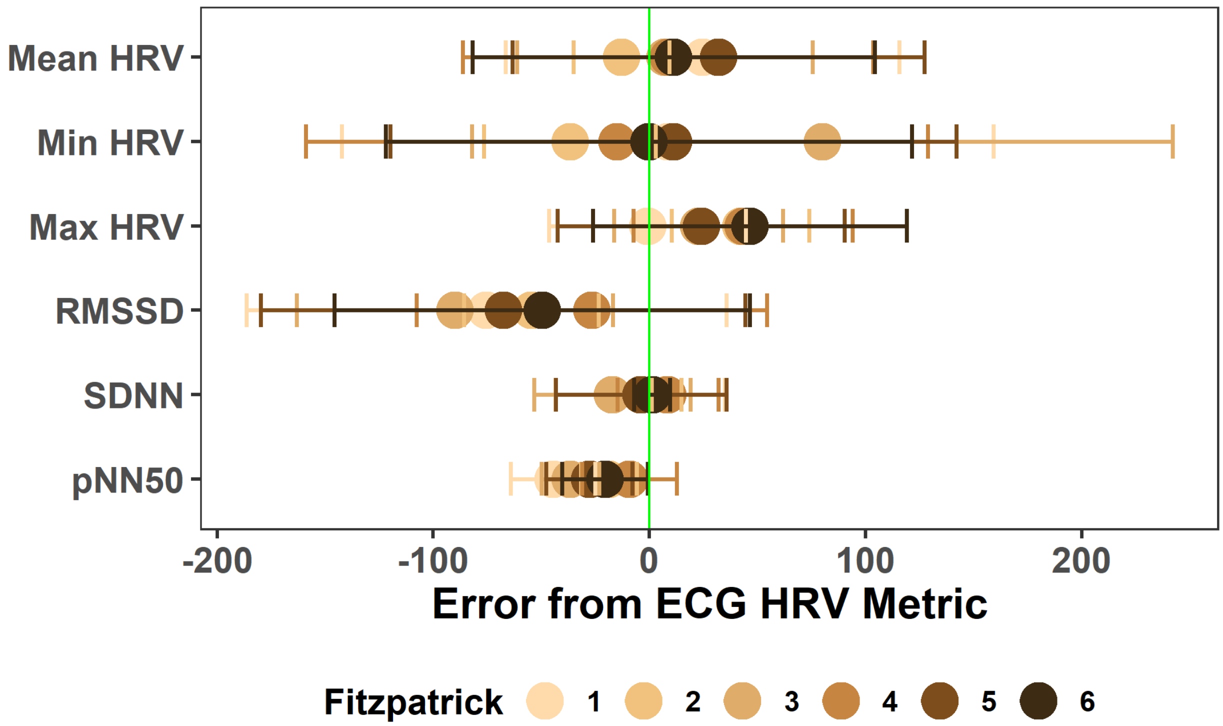

Supplementary Figure 3. *Error in optical HRV time-domain metrics across skin tones. Standard HRV time-domain metrics include: Mean Heart Rate Variability, Minimum Heart Rate Variability, Maximum Heart Rate Variability, RMSSD, SDNN, and pNN50.The green vertical line represents no error (no difference from the true metric of HRV from the ECG).*

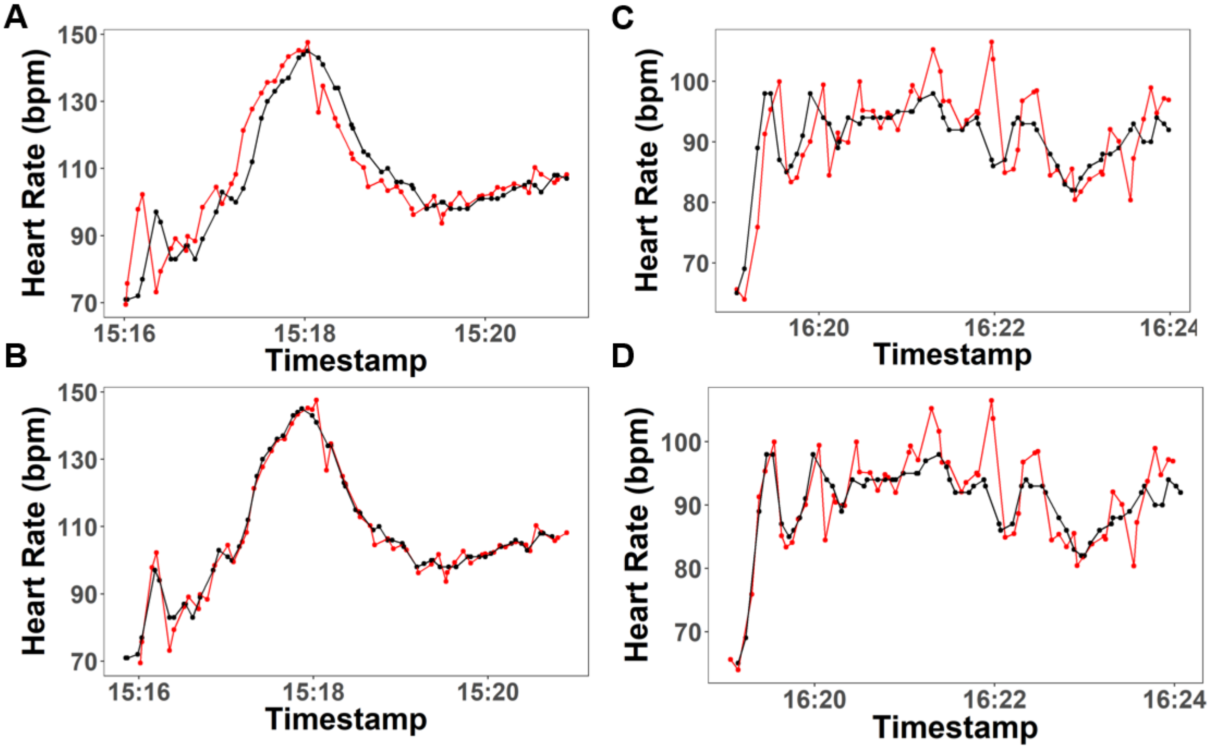

Supplementary Figure 4. (A) *Original Signal, Fixed Lag* ***(B)*** *Lag Correction on Fixed Lag Signal* ***(C)*** *Original Signal, Dynamic Lag* ***(B)*** *Attempted Lag Correction on Dynamic Lag Signal; ECG HR is shown in red and Wearable HR is shown in black.*

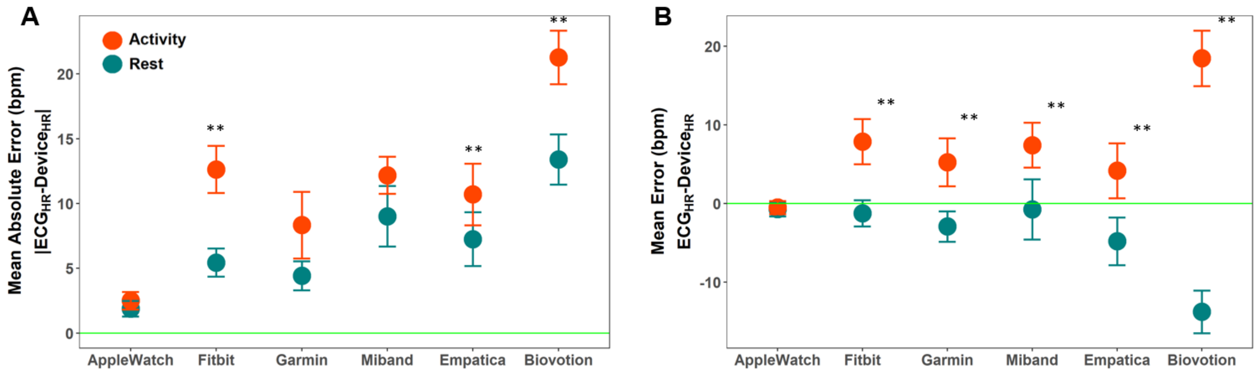

Supplementary Figure 5. *With optimized window length for each participant, device, and activity condition:* ***(A)*** *Mean Directional Error in heart rate (bpm) across devices during rest (teal) and activity (orange) shows the relative differences from the ECG. The green horizontal line represents no error (no difference from the true measurement of HR from ECG). Error bars show the 95% confidence interval.* ***(B)*** *Mean Absolute Error in Heart Rate (bpm) across devices during rest (teal) and activity (orange). This shows the true difference in HR from the ECG but does not show the sign of the difference. The green horizontal line represents no error (no difference from the true measurement of HR from ECG). Error bars show the 95% confidence interval.*

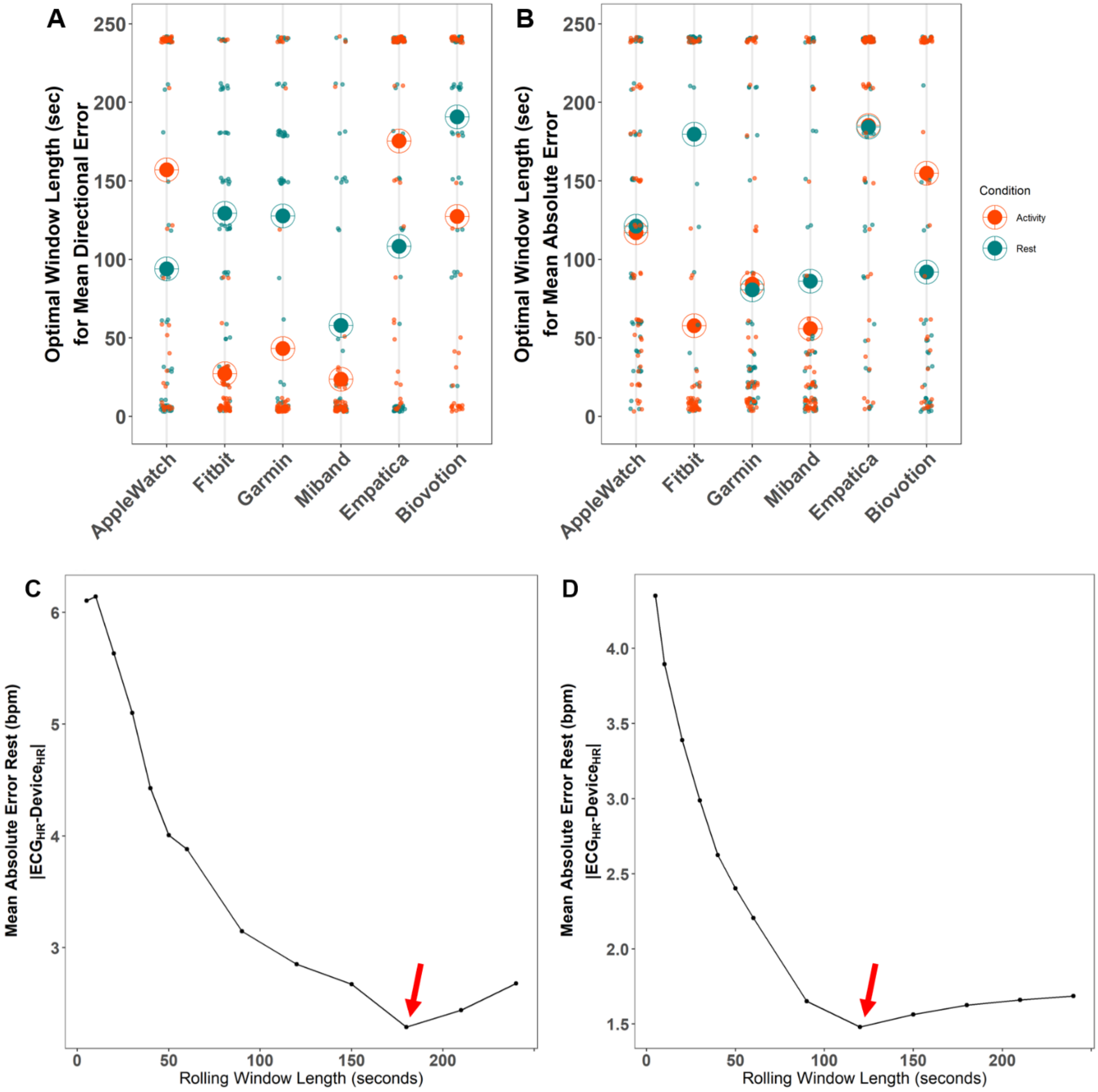

Supplementary Figure 6. *Sensitivity analysis on window length for Mean Directional Error* ***(A)*** *and Mean Absolute Error* ***(B).*** *Points are individual participants; target shapes are mean values for that device over all participants for each condition- rest (teal) and physical activity (orange).* ***(C)*** *and* ***(D)*** *show representative plots of the Mean Absolute Error across rolling windows. Red arrows point to the optimal rolling window length used in the sensitivity analysis.*

Supplementary Table 1. *An analysis of devices combining cost, device release year, market size, target market, and access to raw data during rest and activity (as a percentage of participants) shown in this research study. *The Empatica E4 and Biovotion Everion (only on research application on Android) are the only devices that provide raw PPG signal to users. The Garmin Vivosmart 3 and Xiaomi Miband provide HR data from their native app. Apple Watch 4 and Fitbit Charge 2 require non-native applications to export HR data.*

DEVICE	COST (USD) AT TIME OF STUDY	DEVICE RELEASE YEAR	MARKET SIZE (2018)	TARGET MARKET	ACCESS TO RAW DATA
**APPLE WATCH 4**	$349.00-$459.00	F 2018	46.2 million	Consumer	No*
**FITBIT CHARGE 2**	$129.45-$159.99	F 2016	13.8 million	Consumer	No*
**GARMIN VIVOSMART 3**	$92.73	S 2017	10.5 million	Consumer, active	HR only
**XIAOMI MIBAND 3**	$27.00-$28.98	S 2018	23.3 million	Consumer	HR only
**EMPATICA E4**	$1690.00	S 2017	< 1 million	Research, epilepsy	Yes
**BIOVOTION EVERION**	$499 + annual subscription ($750 startup+ $39/year)	S 2017 (est.)	< 1 million	Medical-grade wearable	Yes*

Supplementary Table 2. *Results of literature review of number of devices being used in research studies in PubMed and ClinicalTrials.gov. Consumer-grade device release year and software version used for the duration of the study are shown for the specific devices used in this study.*

Search Term	Number of studies in PubMed	Number of studies in ClinicalTrials.gov	Device Release Year	Software version (used in this study)
Apple Watch 4	24	5	Fall 2018	5.1.3
Fitbit Charge 2	61	34	Fall 2016	22.55.2
Garmin Vivosmart 3	11	3	Spring 2017	5.10
Xiaomi Miband 3	0	0	Spring 2018	NA
Empatica E4	7	15		-
Biovotion Everion	0	3		-
Apple Watch	68	30		
Fitbit	476	449		
Garmin	108	40		
Miband	1	2		
Xiaomi Miband	0	1		
Empatica	20	22		
Biovotion	4	4		

Supplementary Table 3. *Mean Absolute Error in heart rate (bpm) at rest and during activity for all devices. P-values calculated from paired, two-sided t-tests. ** indicates significant difference in error between baseline and activity with a Bonferroni multiple hypothesis corrected p-value of 0.0042.*

Device	Absolute Error at Rest	Standard Deviation of MAE at Rest	Absolute Error During Activity	Standard Deviation of MAE During activity	P-value Absolute Error
Apple Watch	4.4	2.7	4.6	3.0	0.59
Fitbit	7.3	4.2	12.8	6.8	.0000019**
Garmin	7.0	5.0	9.8	9.2	0.056
Miband	10.2	7.3	13.8	6.1	0.0051
E4	11.3	8.0	12.8	8.5	0.0012**
Biovotion	16.5	6.4	19.8	5.3	0.38
**Mean**	**9.45**		**12.3**		
**Standard Deviation**	**4.2**		**5.0**		

Supplementary Table 4. *Mean Relative Error in heart rate (bpm) at rest and during activity for all devices. P-values calculated from paired, two-sided t-tests. ** indicates significant difference in error between baseline and activity with a Bonferroni multiple hypothesis corrected p-value of 0.0042.*

Device	Directional Error at Rest	Standard Deviation of MDE at rest	Directional Error During Activity	Standard Deviation of MDE during activity	P-value Directional Error
Apple Watch	-0.090	2.8	0.79	2.9	0.21
Fitbit	0.34	5.3	7.6	10.5	.000018**
Garmin	-0.85	6.2	5.5	11.3	.00057**
Miband	-0.064	11.7	7.6	10.3	.000030**
E4	-3.9	12.0	6.3	12.4	.0000043**
Biovotion	-13.6	9.1	18.9	5.6	1.3e-19**
**Mean**	**-3.2**		**7.8**		
**Standard Deviation**	**5.4**		**6.0**		

Supplementary Table 5. *Missingness at rest and during activity for consumer-grade devices.*

	Mean Missingness at Rest (% missing)	Standard Deviation of Missingness at Rest (% missing)	Mean Missingness during Activity (% missing)	Standard Deviation of Missingness during activity (% missing)	P-value paired t-test (activity vs. rest, ** indicated <0.0125)
Apple Watch 4	2.7	9.0	3.4	23.5	0.840
Fitbit Charge 2	18.7	12.0	10.4	13.7	0.00226**
Garmin Vivosmart 3	10.0	20.1	-3.7	24.9	7.47e-07**
Xiaomi Miband 3	5.9	42.7	-14.2	43.5	6.34e-05**

Supplementary Table 6. *Mean Absolute Error in heart rate (bpm) at rest* ***(A)*** *and during activity* ***(B)*** *for all devices across Fitzpatrick skin tones.*

A

	1	2	3	4	5	6	**Mean**	**Standard Deviation**
Apple Watch	4.7	5.3	5.1	3.6	3.1	4.8	4.4	0.88
Fitbit	7.2	7.2	7.1	6.8	8.3	7.1	7.3	0.52
Garmin	6.7	6.0	8.6	5.7	5.6	9.1	7.0	1.5
Miband	11.7	7.9	8.8	9.5	8.8	14.4	10.2	2.4
E4	12.8	15.6	10.4	10.7	8.8	10.4	11.5	2.4
Biovotion	13.8	16.6	15.4	18.1	16.8	18.0	16.5	1.6
**Mean**	9.5	9.8	9.2	9.1	8.6	10.6		
**Standard Deviation**	3.8	5.0	3.5	5.1	4.6	4.8		

B

	1	2	3	4	5	6	**Mean**	**Standard Deviation**
Apple Watch	5.9	5.3	4.7	3.5	3.5	5.3	4.7	1.0
Fitbit	12.1	9.9	10.8	14.7	14.0	15.1	12.8	2.2
Garmin	9.9	9.3	6.2	16.7	7.1	9.6	9.8	3.7
Miband	13.8	12.1	10.3	20.6	14.5	12.8	14.0	3.5
E4	12.4	8.4	10.7	14.0	14.6	15.9	12.7	2.8
Biovotion	18.9	19.4	17.7	18.9	24.0	20.1	19.8	2.2
**Mean**	12.2	10.7	10.1	14.7	13.0	13.1		
**Standard Deviation**	4.3	4.8	4.5	6.0	7.1	5.2		

Supplementary Table 7. *Mean Directional Error in heart rate (bpm) at rest* ***(A)*** *and during activity* ***(B)*** *for all devices across Fitzpatrick skin tones.*

A.

	1	2	3	4	5	6	**Mean**	**Standard Deviation**
Apple Watch	0.25	0.04	-0.58	0.30	0.28	-0.62	-0.056	0.43
Fitbit	3.72	1.68	1.23	-1.92	-1.58	-0.24	0.48	2.15
Garmin	-1.03	0.16	-0.60	-0.81	-0.93	-1.83	-0.84	0.65
Miband	3.43	-3.10	1.60	0.18	-3.47	0.49	-0.15	2.69
E4	0.44	-7.40	-4.08	-1.52	-5.76	-4.15	-3.74	2.83
Biovotion	-10.0	-14.25	-9.18	-17.05	-14.04	-17.32	-13.64	3.43
**Mean**	-0.53	-3.81	-1.94	-3.47	-4.25	-3.95		
**Standard Deviation**	5.00	6.05	4.08	6.71	5.24	6.75		

B

	1	2	3	4	5	6	**Mean**	**Standard Deviation**
Apple Watch	0.57	1.28	1.82	-0.41	0.48	0.88	0.77	0.76
Fitbit	9.17	3.88	3.06	9.51	11.41	8.69	7.62	3.35
Garmin	5.49	3.25	2.14	11.35	3.55	7.31	5.52	3.40
Miband	9.11	9.85	7.55	3.79	5.52	9.59	7.57	2.45
E4	8.53	6.62	1.87	6.58	11.50	4.18	6.55	3.34
Biovotion	18.29	18.42	16.37	18.05	22.81	19.64	18.93	2.17
**Mean**	8.53	7.21	5.47	8.14	9.21	8.38		
**Standard Deviation**	5.81	6.24	5.77	6.40	7.96	6.38		

Supplementary Table 8. *Missingness at rest and during activity for consumer-grade devices across skin tones.*

	Mean Missingness at Rest (% missing)	P-value unpaired t-test (skin tone vs. other skin tones)	Standard Deviation of Missingness at Rest (% missing)	Mean Missingness during Activity (% missing)	P-value unpaired t-test (skin tone vs. other skin tones)	Standard Deviation of Missingness during activity (% missing)
Apple Watch 4						
1	3.73	0.753	8.85	10.22	0.624	39.60
2	-1.36	0.0248	4.01	-1.35	0.206	6.15
3	6.09	0.166	7.92	15.10	0.243	35.6-
4	5.07	0.443	9.96	-3.01	0.0541	1.74
5	2.42	0.941	13.29	4.32	0.902	24.20
6	-1.95E-15	0.226	7.10	-4.34	0.0201	1.28
Fitbit Charge 2						
1	21.00	0.566	10.88	8.51	0.636	10.54
2	17.30	0.739	12.28	4.80	0.229	13.42
3	20.45	0.603	11.68	6.26	0.254	11.90
4	11.34	0.0287	9.40	11.11	0.863	12.58
5	22.28	0.418	14.55	14.48	0.429	16.75
6	19.70	0.778	12.64	16.16	0.207	15.54
Garmin Vivosmart 3						
1	-1.44	0.0561	14.07	-12.61	0.268	20.82
2	4.06	0.266	14.47	-6.47	0.559	11.00
3	15.08	0.229	12.36	4.72	0.250	24.46
4	9.76	0.963	12.89	-6.94	0.626	20.61
5	8.42	0.804	20.09	-6.20	0.714	21.45
6	19.16	0.320	33.42	1.63	0.628	40.20
Xiaomi Miband 3						
1	-20.00	0.122	42.57	-32.11	0.0765	22.59
2	-14.04	0.0762	29.27	-23.42	0.236	16.13
3	18.36	0.0819	15.44	-4.41	0.404	38.84

4	16.51	0.487	49.60	-6.69	0.603	46.64
5	8.64	0.875	57.50	-17.97	0.853	69.81
6	15.71	0.453	44.77	-7.36	0.605	45.61

Supplementary Table 9. *Mixed Effects Comprehensive and Marginal Model Results of ANOVA and Likelihood Ratio Test (interaction model) with Window Length as an effect.*

Mixed Effects Model	Mean Directional Error p-value(***<0.001, **<0.01, *<0.05)	Mean Absolute Error p-value(***<0.001, **<0.01, *<0.05)
Comprehensive Model	<2.20e-16***	<2.20e-16***
Marginal Model: Skin Tone	0.725	0.0901
Marginal Model: Window Length	1.07e-05***	0.00135**
Marginal Model: Condition	<2.20e-16***	1.26e-11***
Marginal Model: Device	0.0383*	<2.20e-16***
Interaction Model: Skin Tone and Window Length (Likelihood ratio test)	0.673	0.184

Supplementary Table 10. *Demographic breakdown of participants in the Skin Tone Equity Project. Broken down by gender, BMI, Fitzpatrick skin tone (1-6), and von Luschan skin tone (1-36). In parentheses are the number of participant results used in the study (53 total), since 3 individuals were excluded from results due to having incomplete ECG records.*

Gender 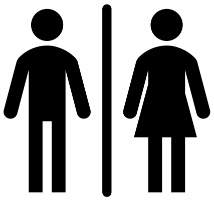	**Female**	**Male**	**Other**						
	32	21	0						
Fitzpatrick Skin Tone 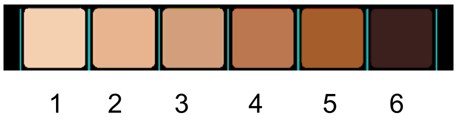	**1**	**2**	**3**	**4**	**5**	**6**			
	7	8	10	9	9	10			
BMI 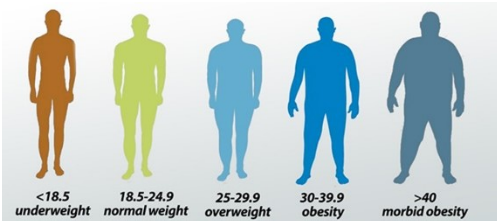	**15-20**	**20-25**	**25-30**	**30-35**	**40+**				
	13	25	11	3	1				
von Luschan Skin Tone 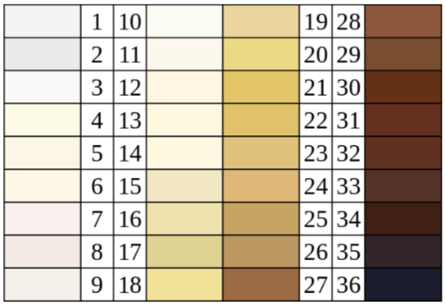	**0-12**	**12-15**	**16-18**	**19-21**	**22-24**	**25-27**	**28-30**	**31-33**	**34-36**
	0	8	0	6	8	11	10	5	4

(Supplementary Table 10 Image sources: BMI source: https://study.com/academy/lesson/what-is-bmi-definition-formula-calculation.html, Fitzpatrick source: https://perfectimage.co/whats-skin-type-fitzpatrick-system/, Von Luschan source: https://commons.wikimedia.org/wiki/File:Felix_von_Luschan_Skin_Color_chart.svg)
